# News and Miscellany

**Published:** 1901-01

**Authors:** 


### News and Miscellany.

**They call** the grippe now so prevalent "Hobensack," because "everybody takes it."

**The Osteopaths** claim that all diseases are caused by dislocated bones. Boneset ought to be a specific.

**Change of Address:**—Dr. J. M. Strayhorn has removed from Bartlett to Waco; Dr. W. H. Seale from Center Point to Marquez, Texas; Dr. M. L. Langford from Baileyville to Corsicana.

**The "Red Back"** begins the new century in fine shape, and will keep up a rattling pace—till next century. No let up on quacks and quackery in or out of the profession. Send along your little dollar for a year's subscription.

**Dr. R. R. Porter** of Columbia, Texas, died at his home November 30, 1900. Dr. Porter was born in Giles County, Tenn., in 1832. He attended lectures in Philadelphia two winters, and, in 1861, he graduated from the Medical Department of the University of Nashville. Dr. Porter practiced medicine in Brazoria County (Columbia and vicinity) forty-five years.

**Good Work by Two Austin Boys.**—An unprecedented record. James Maxwell Loving and Louis Harry Kirk completed the studies of four courses in three years at the University of Texas and were awarded the degree of "B. S." In the meantime they studied medicine, and passing the Freshman's examination, entered the Sophomore class in the Medical Department, Galveston.

**Plague in San Francisco.**—According to the latest weekly report from the M. H. S. (December 28, 1900) there has been but one case of plague in San Francisco since November 4. That occurred December 7; fatal. Total cases, March 7th, 1900, to December 7, 1900, 22. Total deaths, 22. The Surgeon General M. H. S. has kept this record standing; and since November 1, only four cases have been added. Looks like pretty effective work is being done by the M. H. S. in preventing the spread of the disease.

**One of Our Swellest Doctors** of Austin will give society a surprise on 30th, instant, by getting married. He has been the despair of the eligibles for some years, and the engagement has been kept very quiet; only a few of us fellow M. Ds. know anything about it at this date. The doctor and his beautiful bride will go at once to Havana, he to attend the Pan-American Medical Congress.

---

**New Orleans Polyclinic.**—Physicians will find the Polyclinic an excellent means for posting themselves upon modern progress in all branches of medicine and surgery. The specialties are fully taught, particularly laboratory work. Fourteenth annual session opens November 12, 1900. For further information address Dr. Isadore Dyer, Secretary, New Orleans Polyclinic, New Orleans.

---

**The "Red Back"** is usually issued by the tenth of each month; but in consequence of sickness among the printers, and a press of government work, it is late this month. The program of the recent meeting of the Central Texas Medical Association (January 8-9) was put in type and we expected to mail out before the meeting. This is the first time in fifteen years that the JOURNAL has not come up smiling, on time.

---

**Our sprightly contemporary**, the *Texas Courier-Record of Medicine* at Fort Worth, announces its "new editorial staff" as follows: Drs. W. A. Adams, A. C. Walker and E. D. Capps, of Fort Worth, and Dr. J. B. Shelmire, of Dallas. We expect to see some good work by these able gentlemen, and that they will keep the C. R. up to the high standard maintained under the former editorial management. Luck to the C. R.

---

**The Southern Practitioner** (Nashville), current issue, contains a history of the "Osborn Treatment" for the abortion of smallpox, and a biographical sketch of the author, Dr. Thomas Crutcher Osborne, of Cleburne, Texas, together with an excellent picture of the venerable gentleman. The paper is by Dr. Samuel Hollingsworth Stout of Dallas—the Medical Director of the hospitals of the Southern Confederacy during the Civil War. Dr. Osborne was born in Nashville May 4, 1818.

**The Journal** is much gratified to state that the Medical Department of the Texas State University is in full swing, with a large and very promising class of young men. The disaster of September 8th, outside of some damage to the school's property, and the hospitals, which was quickly repaired, seems to have not interfered with the regular work of the college, and we learn that everything is working smoothly, with a larger class of first course students than usual. The graduating class will be smaller.

---

**Astronomical.**—*He had been there.* Said the siren to the young student, as she Saturn her scarlet couch: "Won't you come in and spend an hour with Venus?" "No; thanks," said the young student, "Not any. I've been there. I spent an hour with Venus already last year, and then went up to the hospital and spent six months with Mercury, and since then I have been laid up at Ma's; and don't Juno, I consider it a Sirius matter." Saying which the young student Sidereal big sigh and moved on.

---

**The El Paso Midwinter Carnival** will be held at El Paso January 17th, 18th to 19th (inst.) Special rates on all roads. Big attractions have been provided, and the Carnival has been extensively advertised. The Secretary of the Carnival Association says: "To sum the whole matter up, the Carnival will be an exemplification of life and the people will realize that they are still living and enjoying the fruits of earth. El Paso will certainly put the patent leather finish on any carnival that has ever been seen during the palmy days of a semi-tropical sun.

---

**Ninth Annual Report** S. W. Insane Asylum, San Antonio, Texas, for year ending October 31, 1900. Marvin S. Graves, A. M., M. D., Superintendent. Admitted since last report, 420. On hand last report, 474. Died, 86. Discharged restored, 59. Discharged improved, 29. Discharged not improved, 16. Total, 190. Remaining in Asylum October 31st, 1900, 679. Furloughed, 24. Escaped, 1. Total on hand, 703. The expense of actual maintenance was $76,192.76. Average daily attendance, 615: annual cost *per capita* $123.90.

---

**The New York State Medical Association** has abandoned the publication of a yearly volume of Transactions, and adopted the journal plan. We have received Volume I, No. 1, of *The New York State Journal of Medicine*, January, 1901. Quarto, 24 pages. Contains proceedings of Seventeenth Annual Meeting, New York, October 15, 1900, some Association notes, several original communications, obituary E. R. Squibb, Charter and By-Laws of the Association, etc. No editorial matter; no editor announced. Committee on Publication: Jas. A. Bintenshaw, Chairman, 381 West End Avenue, New York.

---

**Another Western Man** who is attracting much attention in the same school is Dr. I. N. Love, lately of St. Louis. His initial lecture at the New York Post-graduate Medical School was numerously attended; and was warmly commended by his auditors. His assertion that in post-graduate courses medicine is neglected and all attention directed to specialties, particularly surgery and gynecology, struck every one as true; and his plea for a more careful study of clinical medicine, both at the bedside and by laboratory methods, elicited expressions very favorable to the speaker. Dr. Love will have almost unlimited clinical mateterial at his disposal, and with his native eloquence and wit, coupled with his western vigor and enthusiasm, ought to achieve greater distinction in his new field in a brief period than many of his co-laborers have been able to attain by many years of toil. His association with that magnetic teacher and royally good fellow, Dr. Reginald W. Wilcox, ought to spur him to unusual endeavor and assist in bringing out all of the good and practical common sense which is characteristic of the Western practitioner in general, and of this celebrated medical editor in particular.—*American Journal Surgery and Gynecology.*

---

**Medical College Consolidation.**—The important announcement is made that the Marion-Sims College of Medicine and the Beaumont Hospital Medical College of St. Louis have agreed to a consolidation, which will become effective May 1st, 1901. The terms of the consolidation contemplate a utilization of the entire teaching force of the two institutions and a union of their clinical facilities and laboratory equipment.

The old Beaumont Building at Jefferson Avenue and Pine Street will be sold, and the Marion-Sims Building at Grand Avenue and Caroline Street, with new additions already projected, will be used by the new Marion-Sims-Beaumont College of Medicine.

It is manifest that the new institution will have abundant clinical advantages, the following institutions being under the control of members of the new faculty: Alexian Brothers' Hospital, Rebekah Hospital, St. Mary's Infirmary, Josephine Hospital and Grand Avenue Dispensary. In addition, the following hospitals will afford clinical material: City Hospital, St. Louis Insane Asylum, Protestant Hospital and Baptist Sanitarium.

